# The emergence of *Klebsiella pneumoniae* endogenous endophthalmitis in the USA: basic and clinical advances

**DOI:** 10.1186/1869-5760-3-28

**Published:** 2013-02-04

**Authors:** Amir H Kashani, Dean Eliott

**Affiliations:** 1 Associated Retinal Consultants, William Beaumont Hospital, Royal Oak, MI 48067, USA; 2 Massachusetts Eye and Ear Infirmary, Harvard Medical School, Boston, MA 02114, USA

**Keywords:** *Klebsiella pneumoniae*, Endogenous, Endophthalmitis

## Abstract

Endogenous endophthalmitis (EE) is a rare but devastating infection that occurs secondary to seeding of the intraocular cavity from an extraocular focus. Recent reports suggest the increasing prevalence and incidence of *Klebsiella pneumoniae* as a causative organism in Asian countries. Analysis of the largest cohorts published to date suggests that *K. pneumoniae* endogenous endophthalmitis (KPEE) is 10 to 15 times more prevalent than other causes of EE. The incidence of KPEE among patients with systemic *Klebsiella* infection appears to be >100-fold more common than other causes of EE. The exact reason for these observations is not clear, but a number of studies now suggest that *Klebsiella* serotypes K1 and K2 have virulence factors that enhance their survival in diabetic patients and increase their pathogenicity. Here, we report two cases of KPEE in the USA. We also review the recent clinical and basic science literature on the prevalence, incidence, and pathophysiology of this emerging and devastating infection.

## Review

### Introduction

Endogenous endophthalmitis (EE) is a relatively uncommon but severe infection that comprises 2% to 15% of all cases of endophthalmitis [1-3]. It is commonly associated with underlying immunosuppression including diabetes mellitus, cardiac disease, renal insufficiency and malignancy. While a number of organisms have been implicated in EE, *Klebsiella pneumoniae* has been recognized as an increasingly prevalent cause of EE in the Asian population [1,2,4-12]. In addition to numerous case series from Asia, a growing body of basic scientific research has begun to elucidate the pathophysiology of this particular infection. In this case series and review, we report two cases of systemic *Klebsiella* infection that resulted in EE within the USA. We also discuss recent literature that is starting to shed light on the epidemiology and pathophysiology of this condition.

### Epidemiology

The clinical experience with *K. pneumoniae* endogenous endophthalmitis (KPEE) is varied, and comparison of the numerous retrospective studies is difficult due to differences in the methods and reporting criteria [1]. Nevertheless, several large retrospective studies report a surprisingly high number of cases of KPEE among patients with EE (Table 1). Wong et al. reported a series of 27 patients with bacterial EE from Singapore over a 4-year period [6]. Sixty percent of cases were secondary to *K. pneumoniae*, and 48% of these had hepatobiliary sources of infection. Chen et al. reported 74 patients with EE from Taiwan over a 10-year period [5]. Sixty-one percent of cases were secondary to *K. pneumoniae*, and 53% of these cases had liver abscess. Ang et al. reported 113 patients with EE from Singapore over a 21-year period [9]. Sixty-one patients (54%) and 71 eyes had KPEE. These studies demonstrate that among patients with EE in Asia, the prevalence of KPEE is high (54% to 61%; Table 1). In contrast, Jackson et al. reported a series of 21 eyes in 19 patients with EE from England over a 17-year period, and only one case was secondary to *Klebsiella* (5% prevalence) [1]. Similarly, Okada et al. reviewed all cases of EE admitted to the Massachusetts Eye and Ear Infirmary and Massachusetts General Hospital over a 10-year period. They found 28 patients with the diagnosis of EE, and only one case was due to *Klebsiella* (prevalence 3.6%) [3]. Table 1 summarizes these studies.

**Table 1:** Reported prevalence of KPEE among EE in Asian and non-Asian countries

**Study**		**Date range and location**	**Number of EE cases**	**Prevalence of KPEE among EE (%)**
Asian studies	Wong et al. [6]	1994 to 1997; Singapore	27	60
	Chen et al. [5]	1992 to 2002; Taiwan	74	61
	Ang et al. [9]	1986 to 2007; Singapore	113	54
	Average prevalence in Asian countries			58
Non-Asian studies	Jackson et al. [1]	1984 to 2001; England	19	5
	Okada et al. [3]	1980 to 1990; USA	28	3.6
	Average prevalence in western countries			4.3

The reason for this high prevalence in Asia is not clear. Some data suggest that Asian populations have a high prevalence of *Klebsiella* bacteremia and that *K. pneumoniae* is particularly more likely to cause EE than other organisms. Sheu et al. reported a series from Taiwan of 602 patients admitted with *K. pneumoniae* liver abscess over an 18-year period [13]. Forty-two patients (7%) and 53 eyes developed KPEE. Fifty-three percent of these patients had diabetes. Sng et al. reported a case-controlled study of 133 patients with *Klebsiella* bacteremia from Singapore over a 1-year period [4]. KPEE was reported in five (3.8%) of these patients, and two cases were bilateral. All cases with KPEE were associated with liver abscess. Yang et al. reported a series of 200 patients with *K. pneumoniae* liver abscess admitted to a Taiwanese hospital over a 17-year period [14]. Twenty-seven eyes of 22 patients (11%) developed KPEE, and 68% of these had diabetes mellitus. These studies suggest that the incidence of KPEE among patients with systemic *Klebsiella* infection is 3% to 11% (Table 2). This rate of ocular involvement among patients with systemic *Klebsiella* infection is much higher than ocular involvement in other systemic infections. For example, Jackson et al. reviewed 5,859 cases of all-cause bacteremia at St. Thomas Hospital (England) and reported EE in 19 patients (incidence of <0.01%) [1]. Table 2 summarizes these data.

**Table 2:** Incidence of EE and KPEE among cases of systemic infection

**Study**		**Date and location**	**Cases of systemic *K. pneumoniae *infection**	**Incidence of KPEE among *K. pneumoniae *systemic disease**	**Study**		**Date and location**	**Cases of systemic all-cause bacteremia**	**Incidence of EE**
Asian studies	Sheu et al. [11]	1991 to 2009; Taiwan	602	7%	Non-Asian studies	Jackson et al. [1]	1984 to 2001; England	5,859	**<**0.01%^a^
	Sng et al. [4]	2004 to 2005; Singapore	133	3.8%					
	Yang et al. [12]	1994 to 2001; Taiwan	200	11%					
	Average incidence of KPEE in Asian countries			7.3%					

^a^This is the only study available that reports on the prevalence of EE among a cohort of patients with systemic bacteremia.

The incidence and prevalence calculations above suggest that KPEE warrants further investigation. In fact, a number of studies have specifically looked at the pathophysiology of this particular infection. The findings have been revealing about the mechanisms of infection as well as the particularly poor prognosis of this infection versus other causes of endophthalmitis.

### Clinical experience

The classic features of EE include ocular pain, blurred vision, swollen eyelids, injection and chemosis, anterior chamber inflammation, hypopyon, and elevated intraocular pressure. Systemic disease is often advanced, and patients are usually already hospitalized for bacteremia, sepsis, or other systemic complications before the onset of ocular symptoms. There is no consensus on treatment although most studies suggest early and aggressive intervention. A number of studies have reported the visual outcome of KPEE after treatment. Unfortunately, almost all large series and most case reports have very poor outcomes. One case report [15] and one series [16] suggest that very early vitrectomy can preserve vision, but most studies report very poor outcomes regardless of specific interventions.

For example, Yang et al. reviewed the risk factors, clinical features, and visual outcomes in patients with KPEE associated with *K. pneumoniae* liver abscess. Among the 200 patients with *K. pneumoniae* liver abscess, 22 consecutive patients with KPEE were reported during an 8-year period. Twenty-three percent of patients had bilateral involvement, and 68% of these had diabetes. Despite aggressive treatment, final visual acuity of light perception or worse occurred in 89% of patients of which 41% were eventually eviscerated or enucleated [14].

Similarly, Yoon et al. reviewed seven cases of KPEE. Five patients (71%) had diabetes, and four patients (57%) had liver abscess. Eight percent of eyes had hand-motion vision or worse on presentation. After aggressive treatment including pars plana vitrectomy, 70% of eyes had count fingers vision or worse. However, three eyes had vision better than count fingers, and in all eyes, the retina remained attached throughout 6 months of follow-up [16].

We have previously described an atypical case of KPEE [26] that presented with bilateral intraocular inflammation without hypopyon, and systemic evaluation revealed a widely disseminated infection. In the next subsections, we summarize our experience with two additional cases of KPEE with interesting atypical features that merit attention.

#### Atypical case report 1

A 54-year-old Vietnamese man was referred with pain in the right eye. There was no history of trauma or surgery. He had undergone vitreous tap and injection at an outside facility for presumed endophthalmitis. The patient’s past medical history was significant for hypertension. Visual acuity was counting fingers in the right eye and 20/25 in the left eye. There was less than 1 mm of hypopyon in the right eye and dense vitritis. The patient was admitted for presumed EE and underwent immediate vitrectomy. In addition to vitreous inflammation, the superior temporal retina was necrotic. The quadrant of retinitis was demarcated with laser, and intravitreal vancomycin and ceftazidime were administered. Cultures of aspirated vitreous debris grew *K. pneumoniae*. Systemic evaluation revealed *K. pneumoniae* liver abscess. The patient responded well to systemic and ocular therapies. Although this case had a typical presentation and course, it is remarkable in that the final visual acuity in the right eye was 20/30. There are very few reports of such good outcomes associated with KPEE, and to date, the reasons for these outcomes are not clear.

#### Atypical case report 2

A 47-year-old Hispanic man originally from Mexico was referred for evaluation of uveitis of unknown cause. The patient had been living in Los Angeles for the past 24 years. The patient experienced fever and chills 3 weeks prior and subsequently noticed blurry vision in his left eye 2 weeks prior to presentation. The patient denied any previous ocular disease or trauma. The patient was seen by an outside physician and started on oral Bactrim for presumed prostatitis shortly after the onset of his systemic symptoms. The patient was also seen by an ophthalmologist and started on atropine twice a day OS, prednisolone acetate every hour OS, and oral prednisone 30 mg/day for presumed uveitis. Review of systems was significant for 20- to 30-lb weight loss in the month preceding presentation. The patient’s past medical history was notable for hypertension. The patient denied any drug use, smoking, or travel besides a recent trip to Texas, Arizona, and Mexico.

Visual acuity was 20/80 in the right eye and hand motion in the left eye. Intraocular pressures were normal. Examination of the right eye was unremarkable for any acute process. The left eye demonstrated mild conjunctival injection and chemosis, and a 1.5-mm layered hypopyon. Pigmented debris was noted on the anterior lens capsule. The left fundus revealed moderate vitreous haze, some layered vitreous debris inferiorly and multiple large, white retinal lesions involving the macula. This case was remarkable in that ultrasound revealed a subretinal abscess with vitreous strands and debris.

The patient was diagnosed with EE secondary to presumed UTI and he received intravitreal tap and injection of vancomycin, ceftazidime, and voriconazole. He was tapered off the oral steroids and admitted for immediate systemic treatment. Serum glucose levels were elevated (322 mg/dL) as was the hemoglobin A1c (8.7). Other labs were significant including elevated WBC (12.6), elevated CRP (2.5), positive MHA-TP, negative RPR, and negative human immunodeficiency virus (HIV). He was started on intravenous vancomycin and ceftazidime. The patient underwent pars plana vitrectomy and lensectomy with drainage of subretinal abscess. Material from the vitreous and subretinal abscess grew *K. pneumoniae*. Urine culture was positive for *K. pneumoniae*. The patient was discharged on home IV ceftriaxone, oral metronidazole, and oral levofloxacin for 4 to 6 weeks. The left eye ultimately developed light perception vision and a total retinal detachment. While this case has a typical course and outcome for KPEE, it is unusual in that the infection involved a subretinal abscess.

### Pathophysiology

It is well known that patients with EE usually have comorbid immunocompromising diseases such as diabetes, HIV infection, indwelling catheters, renal failure, cardiac disease, malignancy, or immunosuppressive therapy. KPEE occurs secondary to hematogenous dissemination from an extraocular focus (usually liver) and is rarely associated with surgery or trauma. The ‘Endophthalmitis Vitrectomy Study’ reported that only 5.9% of culture-positive cases were due to gram-negative organisms, and none were due to *Klebsiella*[17]. Only one case series that we could find reported *Klebsiella* endophthalmitis secondary to cataract surgery, trauma, or other causes [18].

In addition to host immunosuppression, *Klebsiella*-specific virulence factors have been implicated in the pathogenicity of this organism. *K. pneumoniae* is an enteric, gram-negative bacillus with a polysaccharide capsule. The composition of the cell surface antigens on the capsule are used to classify *Klebsiella* organisms into serotypes. Organisms with capsule serotype K1 or K2 seem to predominate in cases of liver abscess and metastatic spread to other organs including the eye [19,20]. Of the several serotypes (K1, K2, K5, K20, K54, K57, among others) that have been identified, multiple lines of evidence support the increased virulence of organisms with K1 or K2 serotypes. For example, capsular serotypes K1 and K2 inhibit phagocytosis by neutrophils isolated from diabetic patients *in vitro*[21]. In addition, K1 isolates show significantly higher serum resistance and decreased susceptibility to intracellular destruction by neutrophils than other *Klebsiella* serotypes [20]. A single intraperitoneal injection of human neutrophils containing phagocytosed K1 *K. pneumoniae* leads to abscess formation in multiple sites in mice, whereas non-K1 serotypes do not demonstrate this ability [20]. This suggests that there may be a role for capsular phenotype testing in patients with *Klebsiella* bacteremia. Currently, capsular phenotyping is available in the USA, but it is not commonly performed [22-25]. We recently reported one case of bilateral KPEE with widespread disseminated systemic infection with confirmed K1 capsular serotype in the USA (see the ‘Clinical experience’ section) [26]. To our knowledge, no other cases of *Klebsiella* endophthalmitis with verified K1 serotype have been reported in the USA.

The K1 and K2 serotypes are associated with genotypes which may confer some of their virulence. Specifically, the magA gene (mucoviscosity-associated gene A or capsular polymerase Wzy(KpK1) [27]) and rmpA gene (regulator of the mucoid phenotype) have been found in the K1 and K1/K2 serotypes, respectively [28]. The specific roles of these genes in the pathophysiology of endophthalmitis has yet to be determined, but a recent study in mice showed that the hypermucoviscous *K. pneumoniae* associated with the K1 serotype is associated with more rapid development of phthisis, more pronounced inflammation, and poor recovery of retinal function compared with a control strain [28]. A recent study demonstrates that wild-type magA+ and trans-complemented magA organisms grow more rapidly and cause a more profound decrease in ERG a- and b-waves and cellular infiltration in a mouse model than the magA deficient organism [29]. This study is notable in that the organisms lack any hemolytic or proteolytic toxins [29]. Interestingly, wild-type *K. pneumoniae* (magA+) causes a more inflammatory response (as measured by myeloperoxidase assay) than trans-complemented magA organisms, suggesting additional virulence factors in the wild-type organisms or a pathologic difference in the trans-complemented organism [29]. Analysis of various growth media conditioned by *K. pneumoniae* seems to suggest that a secreted factor(s) may also be involved in *Klebsiella* virulence [11,30]. Systemically, the lethal dose of magA-positive strains in a mouse model was approximately 4 to 5 log units lower than isogenic mutants deficient for magA [19,31,32]. Clinically, both the K1 and K2 serotypes have been associated with a hypermucoviscous (HMV) phenotype. This phenotype is defined by the formation of a mucoviscous string greater than 5 mm when a standard bacteriological loop is passed through a colony [28]. It appears that the HMV phenotype overlaps with the K1/K2 serotypes and is significantly associated with bacteremia and invasive infections including meningitis and endophthalmitis [33]. The risk for metastatic spread to the eye or the CNS with primary *Klebsiella* abscess is noted to be 3% to 10% [34,35]; however, at least 50% of patients with *Klebsiella* endophthalmitis have an underlying liver abscess caused by the same bacteria [1]. The poor response of KPEE to aggressive treatment is not well understood. Some evidence suggest that this may be due to poor surveillance and late diagnosis [9]. For example, our previously published case report [26] as well as a few other studies have noted disseminated systemic infection within the first few days of presentation [13,36]. This suggests that widespread infection was already present before ocular involvement in at least some cases. Other factors may include antimicrobial resistance. *K. pneumoniae* is well known for its resistance to traditional and novel antibacterial agents including third-generation cephalosporins, fluoroquinolones, carbapenems, and extended spectrum beta-lactamases.

## Conclusions

The notably high incidence and prevalence of KPEE in Asia has resulted in a number of informative studies reviewed above. The high incidence of KPEE in patients with systemic *K. pneumoniae* infection may provide a model of EE which can facilitate further clinical studies of this infection. These studies in conjunction with basic science studies of the pathophysiology of *K. pneumoniae* infection are allowing focused hypothesis testing of the mechanism of EE. Therefore, further study of KPEE may facilitate better diagnostic and therapeutic management of endophthalmitis in general.

The data available suggest that patients with liver abscess and immunocompromising conditions should be watched closely for signs and symptoms of hematologic spread and EE. These studies suggest a causal and particularly prevalent relationship between systemic *Klebsiella* infection and KPEE. Serotyping for the K1/K2-positive organisms may play an increasing role in determining which patients are at highest risk. This information can potentially lead to screening guidelines or prophylactic treatment. Patients of Asian descent are at highest risk for *Klebsiella*-related EE. However, it is not clear if this is due to a genetic predisposition to infection among Asians or a higher prevalence of virulent *Klebsiella* organisms in Asia. The studies reviewed here suggest the latter possibility since the polysaccharide capsule is protective against phagocytosis and bactericidal serum factors. This report also highlights the occurrence of KPEE in the USA in patients with no known recent travel history to Asia. No course of treatment has been clearly beneficial, but a few cases where immediate, early treatment (within 24 h of presentation) with pars plana vitrectomy and/or intravitreal antibiotics was performed preserved some degree of useful vision [15].

## Competing interests

The authors declare that they have no competing interests.

## Authors’ contributions

AHK wrote the original manuscript. Both AHK and DE collected data, reviewed the manuscript for corrections/revisions, and contributed significantly to the final product. Both authors revised the manuscript. Both authors read and approved the final manuscript.
